# Ataxin-1 is involved in tumorigenesis of cervical cancer cells via the EGFR–RAS–MAPK signaling pathway

**DOI:** 10.18632/oncotarget.21814

**Published:** 2017-10-10

**Authors:** A-Ram Kang, Hyoung-Tae An, Jesang Ko, Eui-Ju Choi, Seongman Kang

**Affiliations:** ^1^ Division of Life Sciences, College of Life Sciences and Biotechnology, Korea University, Seoul 02841, Korea

**Keywords:** ATXN1, cervical cancer, EGFR–RAS–MAPK pathway, tumorigenesis

## Abstract

Ataxin-1 (ATXN1) is a coregulator protein within which expansion of the polyglutamine tract causes spinocerebellar ataxia type 1, an autosomal dominant neurodegenerative disorder. Previously, we reported that ATXN1 regulates the epithelial–mesenchymal transition of cervical cancer cells. In the present study, we demonstrate that ATXN1 is involved in cervical cancer tumorigenesis by promoting the proliferation of human cervical cancer cells. Chromatin immunoprecipitation assays showed that ATXN1 bound to the promoter region within cyclin D1 and activated cyclin D1 transcription, resulting in cell proliferation. ATXN1 promoted cyclin D1 expression through the EGFR–RAS–MAPK signaling pathway. Mouse xenograft tumorigenicity assays showed that ATXN1 downregulation inhibited tumorigenesis in cervical cancer cell lines in nude mice. Human cervical cancer tissue microarrays and immunohistochemical techniques showed that ATXN1 was significantly upregulated in many such tissues. Our results suggest that ATXN1 plays an important role in cervical cancer tumorigenesis and is a prognostic marker for cervical cancer.

## INTRODUCTION

Ataxin-1 (ATXN1) is an evolutionarily conserved, 98-kDa protein involved in transcriptional regulation and cell signaling. Soluble ATXN1 interacts with many proteins, including Capicua, Notch, Sp1, DRD2, WNT and PP2A [1–4]. ATXN1 seems to be also involved in RNA processing owing to its interaction with RBM17 [5, 6]. Moreover, ATXN1 regulates the cerebellar bioenergetics proteome through the GSK3b-mTOR pathway, which is altered in spinocerebellar ataxia type 1 (SCA1), an autosomal dominant neurodegenerative disorder [7]. An expansion of the polyglutamine tract within ATXN1 causes SCA1 [8, 9]. It has been recently reported that ATXN1 regulates the epithelial–mesenchymal transition of cervical cancer cells [10].

Cervical cancer is the second most common cancer in women worldwide. Although the molecular mechanism underlying cellular proliferation and invasiveness in cervical cancer has been widely studied, the prognosis of patients with cervical cancer remains poor [11, 12]. Therefore, identification of new therapeutic targets requires deeper understanding of the molecular mechanisms of cervical cancer progression. The epidermal growth factor receptor (EGFR) has been found to be overexpressed in 70%–90% of cervical cancer cases [13–15]. Moreover, it has been recently shown that EGFR overexpression independently predicts prognosis in patients with cervical cancer, which makes it a potential therapeutic target [16, 17].

EGF is a 6-kDa polypeptide that elicits a number of biological responses, including profound alterations in cellular growth and differentiation, in a variety of normal and tumoral cell types [18, 19]. EGF induces cell proliferation by binding to the prototype transmembrane tyrosine kinase receptors 1 and 2, resulting in the phosphorylation of several protein components. Signaling pathway components activated by EGFR are associated with the development and progression of a number of solid tumors in humans, including those in breast cancer [20]. The Ras-dependent RAF/MEK/ERK1/2 mitogen-activated protein (MAP) kinase signaling pathway is a key regulator of mammalian cell proliferation. Several studies have confirmed that ERK1/2 signaling is essential in the progression of cells from the G0–G1 phase to the S phase during the cell cycle [21–23]. Activation of ERK1/2 MAP kinases is also associated with the stabilization of c-Myc, induction of cyclin Ds, and downregulation of antiproliferative genes during the G1 phase. Furthermore, ERK1/2 signaling promotes cell survival in certain cellular contexts [24].

Cyclin D1 promotes transition from the G1 phase to the S phase during the cell cycle and is involved in hippocampal neurogenesis and adult neural stem cell proliferation [25, 26]. Previous studies have shown that cyclin D1 mRNA levels increase in the cerebellum of both Atxn1^−/−^ and SCA1 knockin mice [27, 28]; this contradicts the results of recent reports showing that cyclin D1 levels decrease in Atxn1^−/−^ and ATXN1[82Q]-expressing hippocampal NPCs [28].

Because the role of ATXN1 in cervical cancer remains unclear, we attempted to investigate it in our present study. We demonstrate that ATXN1 is overexpressed in cervical cancer cells; this overexpression could have been induced by factors such as EGF and that EGF-mediated ATXN1 induction depends on the activation of the EGFR–RAS–MAPK pathway. Our results confirm that ATXN1 plays an important role in cervical cancer cell proliferation, and therefore, it is a potential therapeutic target for treating cervical cancer.

## RESULTS

### ATXN1 promotes the proliferation of human cervical cancer cells

**Figure 1:**
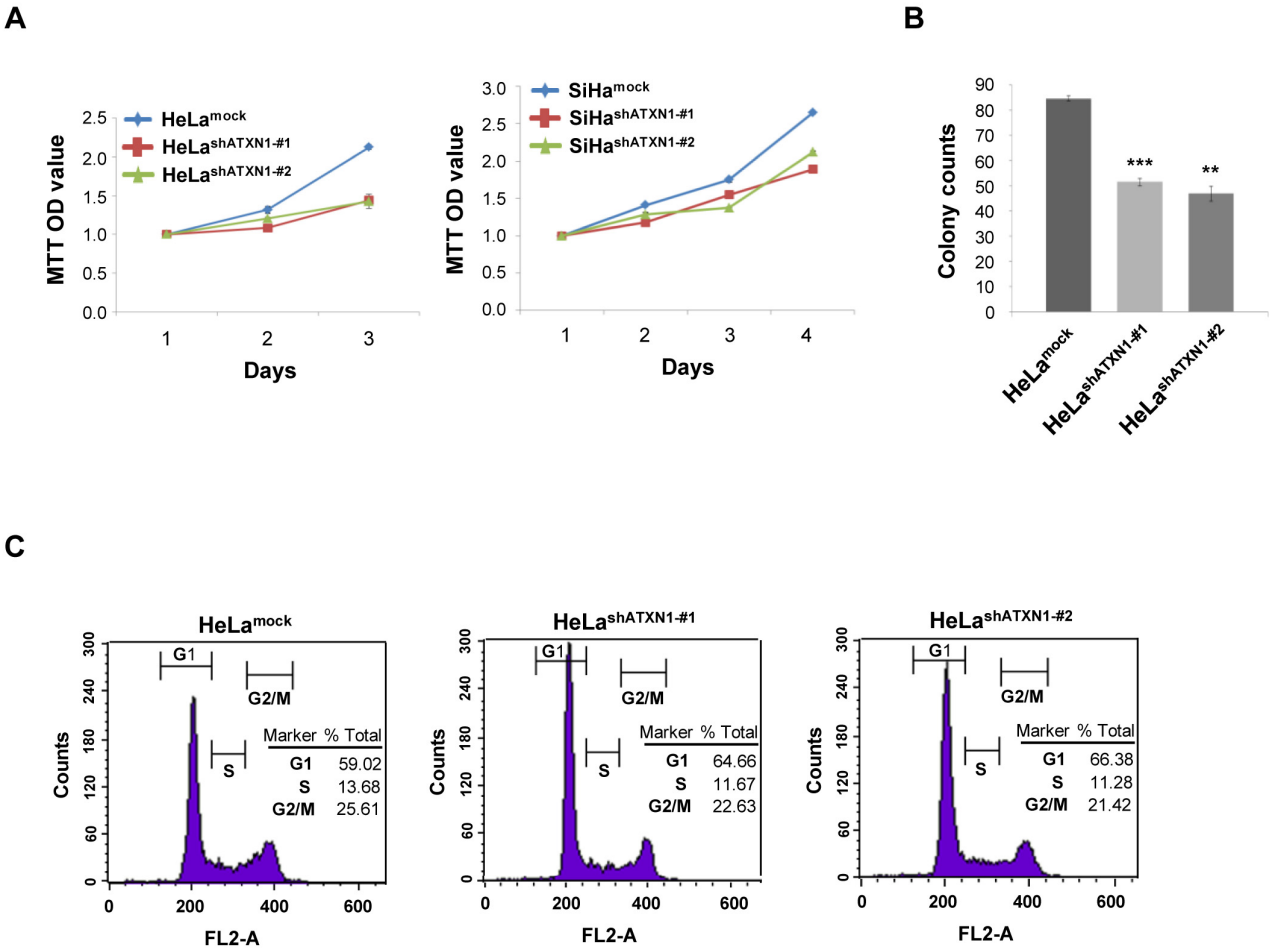
ATXN1 regulates the proliferation of cervical cancer cells **(A)** Left panel: The proliferation rates of HeLa cell lines stably transduced with ATXN1 shRNAs (HeLa^shATXN1-#1^ and HeLa^shATXN1-#2^) were measured using the MTT assay. Right panel: The proliferation rates of SiHa cell lines stably transduced with ATXN1 shRNAs (SiHa^shATXN1-#1^ and SiHa^shATXN1-#2^) were measured using the MTT assay. **(B)** Colony formation of these cells (1 × 10^3^ cells) was monitored for 5 days. The colonies were stained using 0.05% methylene blue, and the colonies in at least five imaging fields were counted. ^*^P < 0.05; ^**^P < 0.01; ^***^P < 0.001 vs. control group (one-way ANOVA). **(C)** Flow cytometry analysis of propidium iodide-treated cells. All data are expressed as the means and S.D. of three independent experiments.

Previously, we reported that ATXN1 plays an important role in the epithelial–mesenchymal transition of cervical cancer cells [10]. In the present study, we further investigated whether ATXN1 also plays a role in tumor growth. We used two cervical cancer cell lines with stably knocked down ATXN1, HeLa^shATXN1-#1^ and HeLa^shATXN1-#2^ cells, that were generated using a lentivirus system and stably expressed ATXN1-specific short hairpin RNAs (shRNAs), as previously described [10]. As a control, a mock lentivirus was tested. We used MTT assays to determine whether ATXN1 knockdown affects the growth rates of the transductants. The assays showed that the inhibition of ATXN1 significantly reduces HeLa cell proliferation in a time-dependent manner (left panel, Figure 1A). In addition, we generated SiHa^shATXN1-#1^ and SiHa^shATXN1-#2^ cervical cancer cell lines and used MTT assays. The experiments showed that the proliferation of SiHa^shATXN1-#1^ and SiHa^shATXN1-#2^ cells also is reduced in a time-dependent manner (right panel, Figure 1A).

To further demonstrate the importance of ATXN1 in cell proliferation, we performed colony-formation assays using HeLa^shATXN1-#1^ and HeLa^shATXN1-#2^ cells and found that each cell line formed fewer colonies compared with the controls (Figure 1B). To determine the mechanism underlying this effect, we performed flow cytometry analysis and found that the number of G1-arrested HeLa^shATXN1-#1^ and HeLa^shATXN1-#2^ cells had increased and that of the S-cells showed a decrease compared with that of the controls (Figure 1C). These results indicate that the knockdown of ATXN1 inhibits the proliferation of cervical cancer cells.

### ATXN1 activates cyclin D1 transcription resulting in cell proliferation

**Figure 2:**
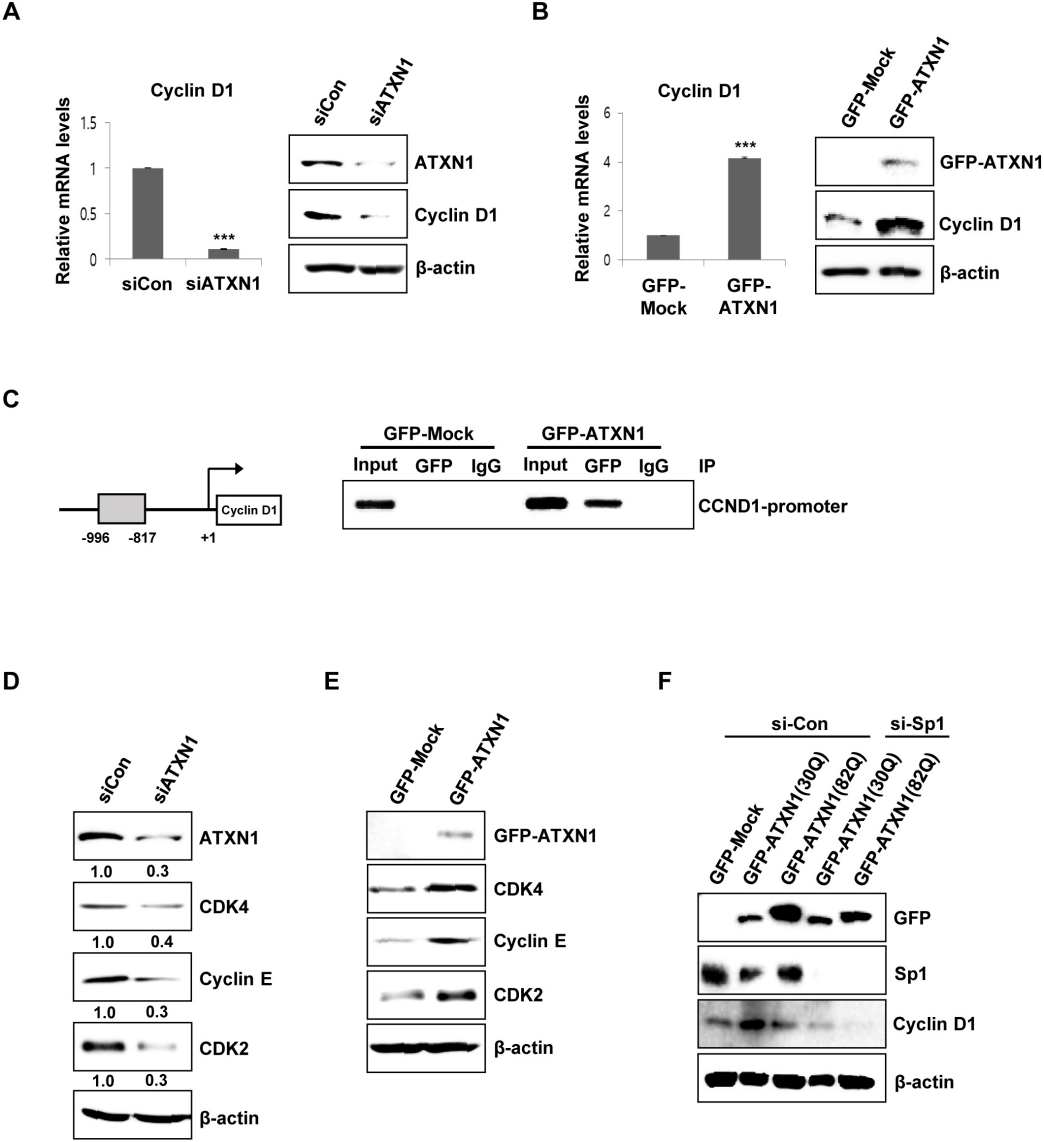
ATXN1-mediated regulation of cyclin D1 and cell cycle-associated proteins **(A)** Left panel: sicontrol (siCon) and siATXN1 were used to transiently transfect SiHa cells, and mRNA levels were estimated using real-time qRT-PCR. Right panel: SiHa cells were transfected with siCon and siATXN1. Cyclin D1 levels were measured 48 h after transfection using western blotting. **(B)** Left panel: GFP-Mock and GFP-ATXN1 were used to transiently transfect SiHa cells, and the mRNA levels were estimated using real-time qRT-PCR. Right panel: SiHa cells transfected with GFP-Mock and GFP-ATXN1. The levels of cyclin D1 were measured after 48 h using western blotting. **(C)** Left panel: Cyclin D1 promoter constructs. Right panel: Chromatin extracts of SiHa cells expressing GFP-Mock and GFP-ATXN1 were immunoprecipitated using an anti-GFP antibody, and the immunoprecipitates were amplified with PCR using cyclin D1 (CCND1) primers. **(D)** SiHa cells were transfected with siCon and siATXN1. The levels of CDK4, cyclin E, and CDK2 were measured 48 h after transfection using western blotting. Densitometry results of protein are shown below each lane. Protein expression was normalized to β-actin levels. Numbers indicate the intensity ratio relative to each control lane (1.0). **(E)** SiHa cells transfected with GFP-Mock and GFP-ATXN1. The levels of CDK4, cyclin E, and CDK2 were measured after 48 h using western blotting. **(F)** Western blotting analysis of SiHa cells transfected with GFP-Mock, GFP-ATXN1(30Q) and GFP-ATXN1(82Q) in the presence or absence of Sp1 siRNA was performed to determine the expression of cyclin D1. IP, immunoprecipitation; CCND1, Cyclin D1. All data were generated from three independent experiments. ^*^P < 0.05; ^**^P < 0.01; ^***^P < 0.001, t-test.

We tried to elucidate the molecular mechanism underlying the cell proliferation related to ATXN1. Because previous studies have demonstrated variations in cyclin D1 mRNA levels in the cerebellum of both Atxn1^−/−^ and SCA1 knockin mice and cyclin D1 is required for progression from the G1 phase of the cell cycle, we first investigated cyclin D1 expression in SiHa cells, a cervical cancer cell line, using ATXN1 knockdown or overexpression vectors. We transfected ATXN1 knockdown expression vectors into SiHa cells and then investigated the expression of cyclin D1 using real-time qRT-PCR and western blotting. The results showed that ATXN1 knockdown decreased cyclin D1 expression and mRNA levels (Figure 2A). We then investigated whether ATXN1 overexpression affected the expression of cyclin D1 in cervical cancer cells. SiHa cells transfected with ATXN1 showed increased cyclin D1 expression and mRNA levels (Figure 2B).

To determine whether ATXN1 directly regulated cyclin D1 gene transcription, we performed ChIP assays and found that ATXN1 expressed by SiHa cells transfected with GFP-ATXN1 was recruited to the proximal region of the cyclin D1 promoter (from −996 to −817 bp) (Figure 2C).

Further examination of G1/S transition-related cell cycle genes by western blotting showed that the knockdown of ATXN1 downregulated the levels of CDK4, CDK2, and cyclin E in SiHa cells (Figure 2D). In contrast, ATXN1 overexpression increased the expressions of CDK4, CDK2, and cyclin E (Figure 2E).

We next examined how ATXN1 enhances cyclin D1 promoter activity. There are numerous transcription-factor binding sites in the cyclin D1 promoter, including the Sp1 binding site [29, 30]. Previously, Goold et al. reported that ATXN1 interacts with Sp1, a zinc finger protein, at the dopamine receptor D2 (Drd2) promoter [4]. SiHa cells containing ATXN1(30Q) or ATXN1(82Q) were treated with control siRNA (si-Con) or Sp1 siRNA (si-Sp1) to knockdown Sp1. The expression levels of cyclin D1 enhanced by ATXN1 were significantly reduced in the presence of si-Sp1 (Figure 2F), suggesting that Sp1 occupies at the cyclin D1 promoter and is involved in the regulation of cyclin D1.

ATXN1 is a transcription regulation protein within which expansion of the polyglutamine tract causes SCA1. We investigated whether polyglutamine length affects cyclin D1 promoter activity. SiHa cells were transfected with wild-type ATXN1(30Q) or mutant ATXN1(82Q) and the expression levels of cyclin D1 were determined in the presence or absence of si-Sp1 (Figure 2F). Cyclin D1 promoter activity is stronger in SiHa cells expressing ATXN1(30Q) than in SiHa cells expressing ATXN1(82Q).

These results indicate that ATXN1 promotes the growth of cervical cancer cells through the upregulation of cyclin D1 expression, which is required for progression from the G1 phase of the cell cycle.

### ATXN1 promotes cyclin D1 expression through the EGFR–RAS–MAPK signaling pathway

**Figure 3:**
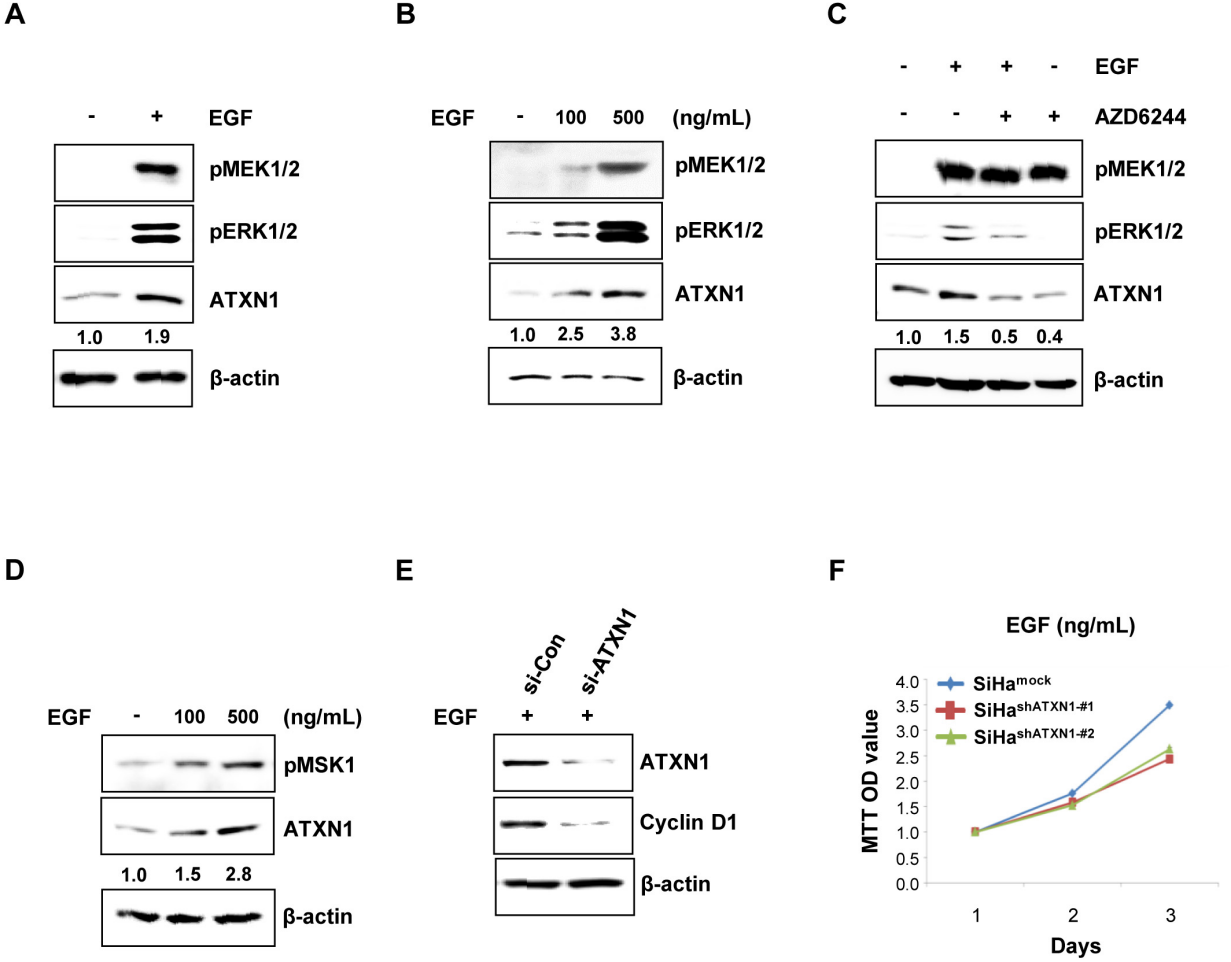

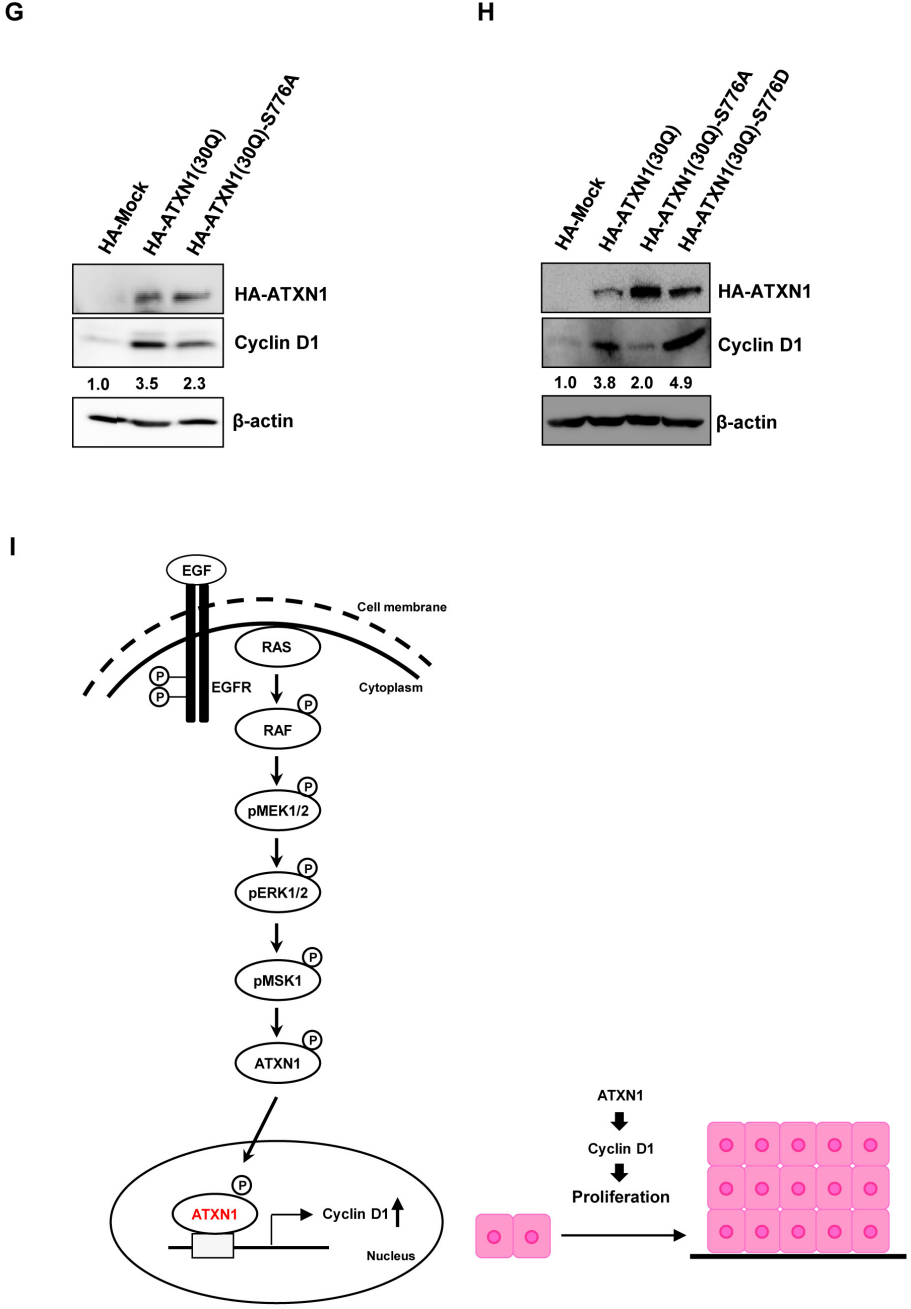
Upstream EGFR–RAS–MAPK pathway components regulate ATXN1 levels **(A)** The expression level of ATXN1 under the presence (+) and absence (−) of EGF, as examined by western blotting. ATXN1 levels were upregulated upon EGF stimulation along with the upregulation of pMEK1/2 and pERK1/2. β-actin was used as the loading control. **(B)** SiHa cells were treated with 0, 100, and 500 ng/mL of EGF for 48 h. Total lysates were harvested and then western blotting was performed using the indicated antibodies. **(C)** SiHa cells were starved in 0.1% FBS for 4 h before treatment. The cells were grown in RPMI medium containing EGF (500 ng/mL) and AZD6244 (1.5 μM) alone for 48 h. EGF + AZD6244: SiHa cells were pretreated with AZD6244 for 1 h prior to the addition of EGF. Untreated control cells were maintained in RPMI with DMSO. ATXN1, pMEK1/2, and pERK1/2 were detected by western blotting. **(D)** SiHa cells were treated with 0, 100, and 500 ng/mL EGF for 48 h. Total lysates were harvested for western blotting using the indicated pMSK1 and ATXN1 antibodies. **(E)** SiHa cells were transfected with siCon or siATXN1. SiHa cells containing siCon and siATXN1 were treated with EGF in RPMI for 48 h. Total lysates were harvested and then western blotting was performed using the indicated antibodies. **(F)** The proliferation rates of SiHa cell lines stably transduced with ATXN1 shRNAs (SiHa^shATXN1-#1^ and SiHa^shATXN1-#2^) were measured using the MTT assay. SiHa^shATXN1-#1^ and SiHa^shATXN1-#2^ cell lines were treated with EGF at the indicated times for 72 h. **(G)** SiHa cells were transfected with HA-ATXN1 or HA-ATXN1(S776A). The levels of cyclin D1 were measured after 48 h using western blotting. **(H)** SiHa cells were transfected with HA-ATXN1 or HA-ATXN1(S776D). The levels of cyclin D1 were measured after 48 h using western blotting. All data are expressed as the means and S.D. of three independent experiments. Protein expression was normalized to β-actin levels. Numbers indicate the intensity ratio relative to each control lane (1.0). **(I)** Left panel: The hypothesized model of ATXN1 signaling involved in the EGF-induced increase in cell proliferation. EGF activates receptor tyrosine kinase, which stimulates Src kinase. Receptor tyrosine kinases, including EGF receptors, signal through their activation of RAS and downstream cascade RAF, MEK1/2, and ERK1/2 to regulate cell proliferation. Finally, these molecules may induce ATXN1, which increases cell cycle regulatory proteins. Right panel: ATXN1 promotes growth of cervical cancer cells by upregulating cyclin D1 during the early stages of tumor development, which in turn promotes the growth of cervical cancer cells that subsequently form a tumor.

To identify the molecular mechanism underlying cell growth in cervical cancer promoted by ATXN1, we analyzed the relevant regulatory signaling pathways. EGF induces cell growth in human cancer cells [19] and activates the Akt and ERK signaling pathways [31, 32]. Considering this, we hypothesized that ATXN1 is involved in EGF-mediated cancer growth. To test our hypothesis, we treated SiHa cells with 500 ng/ml of EGF for 48 h and then measured the ATXN1 levels. ATXN1 levels significantly increased upon EGF stimulation. Furthermore, the phosphorylation levels of MEK and ERK were significantly upregulated after stimulation with EGF (500 ng/ml) in SiHa cells compared with their levels in the control cells (Figure 3A).

EGF experiments were also performed in a dose-dependent manner to verify the induction of ATXN1 upon EGF stimulation. As shown in Figure 3B, stimulation with increasing concentrations of EGF (100–500 ng/ml) resulted in a progressive and sustained activation of ATXN1 levels. MEK1/2 and ERK1/2 phosphorylation were also induced by EGF (Figure 3B). In addition, we assessed the effect of the MEK1/2 kinase inhibitor AZD6244 on the EGF-induced stimulation of ATXN1. This MEK inhibitor was used at a concentration of 1.5 μM, which sufficiently inhibited ERK1/2 phosphorylation in the SiHa cell line. When cells were incubated with both EGF and AZD6244, both ATXN1 levels and ERK phosphorylation decreased (Figure 3C). These inhibitory AZD6244 concentrations are similar to those reported previously for ERBB3/PI3K/AKT signaling in cancer cells [33].

We also investigated whether the observed effect of ATXN1 stability is mediated by MSK1. As shown in Figure 3D, stimulation with increasing concentrations of EGF (100–500 ng/ml) increased ATXN1 levels and MSK1 phosphorylation was also induced by EGF (Figure 3D).

In addition, we investigated whether ATXN1 knockdown still decreases cyclin D1 expression in the presence (Figure 3E) as well as absence (Figure 2A) of EGF. We treated SiHa cells containing siCon or siATXN1 with 500 ng/ml of EGF for 48 h and then measured the cyclin D1 levels. As shown in Figure 3E, the knockdown of ATXN1 decreased the levels of cyclin D1 in SiHa cells enhanced in response to EGF treatment. We also examined whether ATXN1 knockdown affects the growth rates of the SiHa^shATXN1-#1^ and SiHa^shATXN1-#2^ in the presence of EGF. MTT assays revealed that the EGF-induced SiHa cell proliferations were reduced by the knockdown of ATXN1 expression (Figure 3F).

Furthermore, we checked whether the observed effect of ATXN1 on cyclin D1 levels is mediated by phosphorylation at Ser776 in ATXN1. We found a decreased level of cyclin D1 in SiHa cells with ATXN1 (S776A) compared with that in those with wild-type ATXN1 (Figure 3G). In contrast, the potentially phospho-mimicking amino acid substitution S776D increased the expressions of cyclin D1 (Figure 3H).

Our data indicate that ATXN1 promotes cyclin D1 expression through the EGFR–RAS–MAPK signaling pathway, resulting in the proliferation of cervical cancer cells, as shown in Figure 3I, and that EGF is a potent inducer of ATXN1, pERK1/2, pMEK1/2, and pMSK1 in cervical cancer cells.

### ATXN1 downregulation inhibits tumor progression in nude mice with cervical cancer

**Figure 4:**
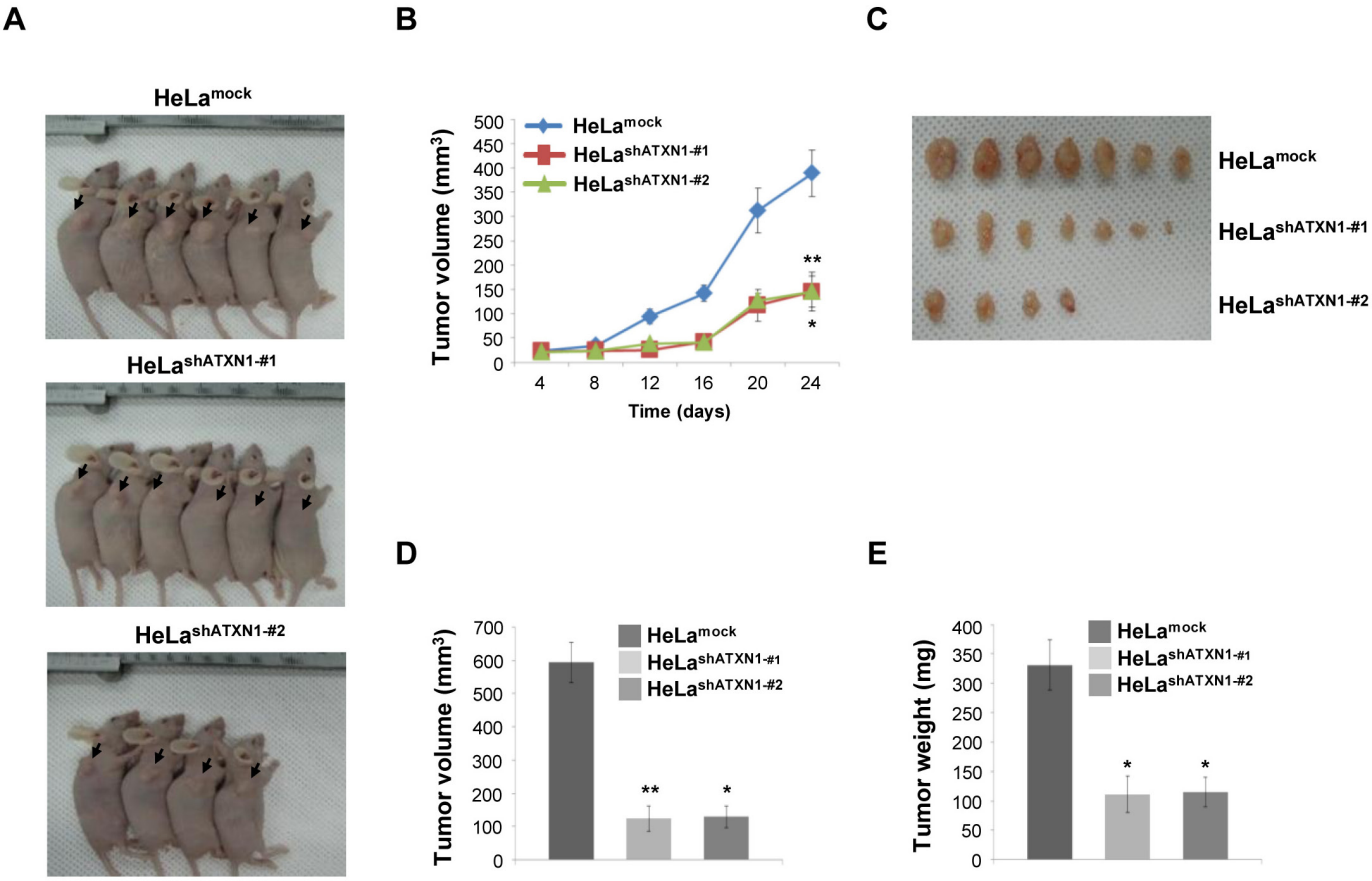
Tumor induction in mice HeLa^shATXN1-#1^ and HeLa^shATXN1-#2^ cells **(A)** HeLa^shATXN1-#1^ and HeLa^shATXN1-#2^ cells were injected into the right flanks of BALB/c nude mice. Images of representative mice from each group are presented to show the tumor volumes (arrows). **(B)** Tumor volumes were measured every 4 days for up to 24 days. ^*^P < 0.05; ^**^P < 0.01; ^***^P < 0.001, t-test. **(C** and **D)** On day 24, tumors were excised from the animals and tumor sizes were measured. ^*^P < 0.05; ^**^P < 0.01; ^***^P < 0.001 vs. control group (one-way ANOVA). **(E)** Tumor weights. ^*^P < 0.05; ^**^ P < 0.01; ^***^P < 0.001 vs. control group (one-way ANOVA). The experimental groups are comprised of 11 and 12 mice engrafted with the control and ATXN1-transduced HeLa cell lines (HeLa^shATXN1-#1^ and HeLa ^shATXN1-#2^), respectively.

To assess the role of ATXN1 in cell proliferation, we established a mouse xenograft model by subcutaneously injecting HeLa^shATXN1-#1^, HeLa^shATXN1-#2^, or control cells into the right flanks of athymic BALB/c nude mice. All the mice produced visible tumors of different sizes 24 days after injection (Figure 4A and 4B). However, the growth of tumors induced by HeLa^shATXN1-#1^ and HeLa^shATXN1-#2^ cells was significantly reduced during the 24-day experiments (Figure 4A and 4B). All mice were sacrificed on day 24, and the tumors were dissected. The growth of tumors in HeLa^shATXN1-#1^ and HeLa^shATXN1-#2^ cells decreased compared with that of those in the control cells (Figure 4C). Further investigation showed that knockdown of ATXN1 expression caused significant reduction in tumor volume (Figure 4D) and weight (Figure 4E) compared with controls. Taken together, these results imply that ATXN1 increased cervical cancer cell tumorigenesis *in vivo*.

### Upregulation of ATXN1 is observed in many human cervical cancer tissues

**Figure 5:**
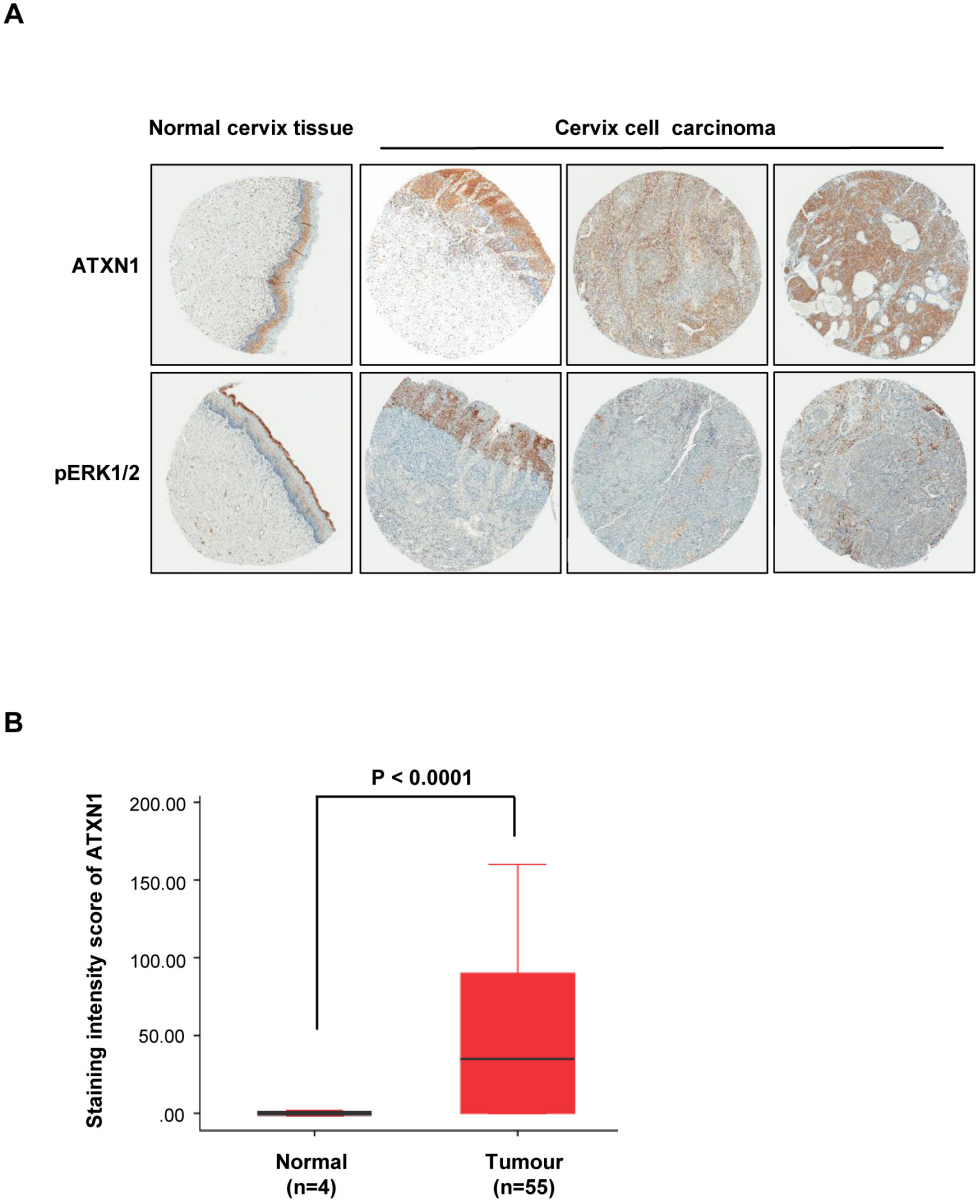
Histological analysis of human cervical cancer tissues **(A)** Immunohistochemical (IHC) analysis of tissue microarrays prepared from human cervical cancers tissues were treated with an antibody against ATXN1 and pERK1/2. Representative samples of ATXN1 and pERK1/2 expression in normal tissues and cervical cancer tissues are shown. **(B)** The correlation plot of H-score quantification for ATXN1. Relative ATXN1 expression levels in cervical tumours (n=55) were compared with that of normal tissues (n=4). Box and whiskers indicate minimum to maximum percentiles in this figure: center line, median value; upper box limit, 75% percentile; lower box limit, 25% percentile; whiskers, minimum or maximum values. Values are means ± SD. P-values were obtained using independent t-test to compare ATXN1 expression between the cervical uterine and cervical carcinoma groups.

We performed immunohistochemical analysis to determine the expression patterns of ATXN1 and pERK1/2 in tissues and human cervical tumors. For this purpose, we analyzed 55 and 4 specimens derived from cervical tumors and normal cervical tissues, respectively. The analyses revealed that the expression of ATXN1 increased in many of the cervical cancer specimens and correlated with pERK1/2 expression (Figure 5A). However, ATXN1 and pERK1/2 staining in normal cervical tissues was consistently lower than that in cervical cancer samples. To clarify the positive correlation between ATXN1 levels in clinical cervical tumors and evaluate their levels in a tumor tissue microarray, including 55 clinical cervical tumor samples, we used TMAlab (Image Scope Software; Aperio Technologies) that generates a numeric score for protein expression for each biomarker, producing an H-score [34] for each marker and each tissue spot. This quantification method has been used to assess the staining intensity of lung, ovarian, and prostate cancers [34] and is a fully automated method that reduces human bias. Expression of ATXN1 was scored according to the staining intensity and proportion of signals in each sample (Figure 5B). Results from a tumor tissue microarray analysis revealed that the ATXN1 expression level was highly elevated in cervical tumor specimens (Figure 5B).

## DISCUSSION

We confirmed that ATXN1 is involved in tumor growth in cervical cancer cells. We performed colony-formation assays using HeLa^shATXN1-#1^ and HeLa^shATXN1-#2^ cells and found that each cell line formed fewer colonies than the control cells. Furthermore, in our *in vivo* studies, we observed a marked decrease in tumor xenograft growth in mice with ATXN1 knockdown. We further found that ATXN1 knockdown could inhibit cervical cancer cell growth and tumorigenesis. Our results indicate that ATXN1 is an important regulator in the progression of cervical cancer. Overexpression and downregulation of ATXN1 promoted and impeded the proliferation of human cervical cancer cells, respectively, implying that ATXN1 may function as an oncogene. The present study provides new insights into the role of ATXN1 in the pathogenesis of cervical cancer.

We identified the cyclin D1 gene as a downstream target of ATXN1 in cervical cancer cell lines. Cyclin D1 is a critical regulator of the G0/G1 to S transition of the cell cycle [35], and its identification here as a downstream target of ATXN1 is consistent with the effect of ATXN1 on promoting the G0/G1 to S transition. We also showed that ATXN1 regulated cyclin D1 expression and transcriptional activities (Figure 2A, 2B, and 2C). Together, these results confirm that ATXN1 promotes the proliferation and malignant phenotype of cervical cancer cells by regulating cyclin D1.

Recently, some studies have shown that several modulators of ATXN1 levels belong to the single RAS–MAPK–MSK1 signaling pathway [36]. The availability of several additional modulators of ATXN1 in the RAS–MAPK–MSK1 pathway may provide further opportunities to explore the development of combination therapeutics. EGF can activate the RAS–RAF–MEK–ERK pathway, and high levels of EGF and constitutive activation of the RAS–RAF–MEK–ERK pathway are observed in cancers [37, 38]. Our results confirm that the EGF pathway is involved in the expression of ATXN1 in cervical cancer. The EGF–RAS–RAF–MEK–ERK pathway is frequently activated in various cancers, including breast cancer, and plays a critical role in tumor growth [20]. We found that under the stimulation of EGF, the RAS–MAPK pathway could activate ATXN1, which plays an important role in tumor growth (Figure 3A and 3B).

Thus, we found that ATXN1 promotes the proliferation and malignant phenotype of cervical cancer cells by regulating cyclin D1. Previously, we discovered a novel mechanism through which ATXN1 regulates the epithelial–mesenchymal transition of cancer cells. Hypoxia induces the expression of the Notch intracellular domain (NICD), which decreases the expression of ATXN1 through ubiquitination and degradation associated with MDM2.

Taken together, we propose a model for the role of ATXN1 in the development of cervical cancer cells as follows: ATXN1 promotes the growth of cervical cancer cells through the upregulation of cyclin D1 during the early stages of tumor development. Hypoxia is a characteristic feature of locally advanced solid tumors, and upregulation of NICD expression induced by hypoxia may decrease ATXN1 expression. Therefore, ATXN1 expression is decreased during the late stages of tumor development. Downregulation of ATXN1 induces the epithelial–mesenchymal transition in cervical cancer cells. ATXN1 knockdown also promotes tumor cell migration and invasion. Metastatic cancer cells survive under normoxia, which increases ATXN1 levels. Thus, ATXN1 promotes the growth of cervical cancer cells and the subsequent formation of tumors through the upregulation of cyclin D1.

We found that ATXN1 enhances cyclin D1 promoter activity and Sp1 among various transcriptional factors increases the promoter activity, indicating that both ATXN1 and Sp1 are involved in the regulation of cyclin D1 transcription (Figure 2F). Currently, we do not know whether ATXN1 directly binds to Sp1. Previously, it was reported that ATXN1 occupies at the dopamine receptor D2 (Drd2) promoter and interacts with Sp1 through the AXH domain of ATXN1 to co-regulate Drd2 expression [4]. Therefore, we believe that the direct interaction between ATXN1 and Sp1 could occur in the cyclin D1 promoter. Further investigation of the transcriptional complexes at the cyclin D1 promoter is required.

Our tissue microarray analysis showed that the expression of ATXN1 is upregulated in cervical squamous cell carcinoma, which represents the most common type of cervical cancer. The data also provided new insight into the prognosis of cervical tumorigenesis and the individualized therapy of patients. Therefore, ATXN1 functions as a proto-oncogene, and its increased expression is activated through the EGFR–RAS–MAPK pathway, exacerbating the malignancy. Together, our findings indicate that ATXN1 may play a role in the proliferation of cervical cancer cells as well as the growth of tumors. Therefore, the inhibition of ATXN1 and ATXN1-mediated activation of the EGFR–MAPK signaling pathway may play an important role in the treatment of cervical cancer.

## MATERIALS AND METHODS

### Cell culture and transfection

The cervical cancer SiHa cells were purchased from the American Type Culture Collection (Manassas, VA, USA) and maintained in an incubator using an RPMI-1640 medium supplemented with 10% fetal bovine serum (Hyclone) at 37°C with 5% CO_2_. Stable HeLa cells were also maintained using Dulbecco’s modified Eagle’s medium (DMEM) supplemented with 10% fetal bovine serum (Hyclone) in an incubator at 37°C with 5% CO_2_. The cells were transfected with siRNA using the Lipofectamine 2000 transfection reagent (Invitrogen, USA) according to the manufacturer’s instructions.

### Plasmids, reagents, and antibodies

The siGENOME SMARTpool contains a mixture of four SMART selection-designed siRNAs (siATXN1) that target the gene encoding ATXN1 and a mixture of four SMART selection-designed siRNAs (siSp1) that target the gene encoding Sp1 (Dharmacon, Lafayette, CO, USA). The pLKO.1-shATXN1 plasmid was constructed as previously described [10]. The HA-tagged ATXN1(30Q)-S776D were generated by making a point mutation in the original construct (ATXN1(30Q)-S776). Human EGF (hEGF) was purchased from R&D Systems (Minneapolis, MN). AZD6244 was purchased from Selleck Chemical (Houston, TX, USA). Primary antibodies targeting the following proteins were used: ATXN1, pERK1/2, pMEK1/2, and pMSK1 (Cell Signaling Technology, USA) and GFP, CDK2, CDK4, Cyclin E, Cyclin D1 and Sp1 (Santa Cruz Biotechnology, USA).

### Immunoblot analysis

SiHa cells were separately transfected with GFP-Mock and GFP-ATXN1(30Q) that contains 30 glutamines in ataxin-1. After 48 h of transfection, the cells were harvested and lysed on ice for 30 min in RIPA buffer supplemented with protease inhibitors (20 mM Tris-Cl, 150 mM NaCl, 0.1% SDS, 1% Triton X-100, and 1% sodium deoxycholate, pH 7.5). Immunoblotting experiments were performed as previously described [39].

### Quantitative real-time PCR

Quantitative real-time PCR was performed according to the method previously described [40]. In brief, SiHa cells were transfected with DNA or siRNA, as indicated in Figure 2A and 2B. After 48 h, total RNA was isolated from the transfected SiHa cells using TRIzol reagent (Invitrogen, USA). The PCR reactions were used to synthesize cDNA fragments from 1 μg of total RNA with the First-Strand Synthesis System (Invitrogen, USA) and an oligo(dT)_20_ primer. Cyclin D1 and GAPDH cDNAs were amplified using the SYBR Green Real-time PCR Master Mix and LightCycler 480 (Roche, Basel, Switzerland).

### Chromatin immunoprecipitation

ChIP assays were performed as previously described [41, 42]. In brief, SiHa cells were transfected with 3 μg of GFP-ATXN1 and empty vector DNAs and incubated for 48 h. DNA was extracted with phenol, precipitated from the aqueous phase using ethanol, and amplified by PCR using cyclin D1-specific primers to detect the human cyclin D1 promoter region, as previously described [43] (primer sequences: 5′-CCGACTGGTCAAGGTAGGAA-3′ and 5′-CCAAGGGGGTAACCCTAAAA-3′). The PCR cycling conditions were as follows: 95°C for 5 min and 35 cycles at 94°C for 20 s, 56.9°C for 20 s, 72°C for 20 s, and 72°C for 5 min. The amplified DNA was electrophoresed on a 2% agarose gel and was visualized using ethidium bromide.

### Cell proliferation and cell cycle analyses

Cell proliferation was assessed using the 3-(4,5-dimethylthiazol-2-yl)-2,5-diphenyltrazolium bromide (MTT; Promega) assay according to the manufacturer’s instructions. In brief, cells at a density of 1 × 10^3^/ml were seeded into each well of a 96-well culture plate and cultured for 24, 48, or 72 h at 37°C. Then, MTT (5 mg/ml in PBS) was added, and the cells were further incubated for 3 h. The water-insoluble formazan that formed was solubilized by adding DMSO and incubating further for 30 min, and the solubilized amount was determined by measuring absorbance at 570 nm using a microplate reader (Bio-Rad Laboratories); cell viability was calculated using the following formula and was expressed as a percentage: (viability of the experimental samples/viability of control samples) × 100. At least three independent experiments were performed. For cell cycle analyses, HeLa cells stably transduced with lentivirus-encoded ATXN1-specific shRNAs (HeLa^shATXN1-#1^ and HeLa^shATXN1-#2^) were fixed in 70% ethanol at 4°C for at least 4 h. The cell cycle analyses were performed according to the method previously described [44].

### Colony-formation assay

Cells were cultured in 12-well plates (1 × 10^3^ cells/well) in 10% fetal bovine serum containing DMEM. After incubation for 5 days, the cells were stained with methylene blue, and the number of colonies was averaged. Colony-forming rates in five imaging fields were determined, and the experiments were performed in triplicates.

### Xenograft tumorigenicity assays

Logarithmically growing HeLa cells were harvested and washed twice with PBS, and 3 × 10^6^ cells in 0.1 ml PBS were then injected subcutaneously into the right upper back region of 6-week-old BALB/c nude female mice. After 24 days, the tumor-bearing mice were sacrificed, and their tumors were measured six times using a caliper. The experimental groups comprised 11 and 12 mice engrafted with the control and ATXN1-transduced HeLa cell lines (HeLa^shATXN1-#1^ and HeLa^shATXN1-#2^), respectively. Tumor volume = 1/2 (width^2^ × length). All experiments with mice were approved by the Institutional Animal Care and Use Committee of the Korea University, Seoul, South Korea.

### Tissue microarrays (TMA) and immunohistochemistry

Human cervical cancer tissue arrays (CZA2) were purchased from SUPER BIO CHIPS. Tissue microarray slides were deparaffinized, rehydrated, and heated for antigen retrieval before incubation with the antibodies. The slides were incubated with an anti-ATXN1 antibody for 1 h at room temperature, treated with a broad-spectrum secondary antibody conjugated with HRP for 1 h, developed using DAB, and mounted after counterstaining with hematoxylin.

### Statistical analysis

Differences between the various experimental groups were calculated using the two-tailed Student’s *t*-test and one-way ANOVA. P-value of <0.05 was considered statistically significant.
